# Supplementary material for: Determinants of Systemic SARS-CoV-2-Specific Antibody Responses to Infection and to Vaccination: A Secondary Analysis of Randomised Controlled Trial Data
Source: Vaccines (Basel). 2024 Jun 20;12(6):691. doi: 10.3390/vaccines12060691 (PMC11209274; doi:10.3390/vaccines12060691)
Supplement: Supplementary file 1 [file vaccines-12-00691-s001.zip › vaccines-3040160 - Vaccines Supplementary Methods and Results.pdf]

SUPPLEMENTARY METHODS AND RESULTS

Determinants of systemic SARS-CoV-2-specific antibody responses to infection and to vaccination: a secondary analysis of randomised controlled trial data

Authors: Juana Claus, Thijs ten Doesschate, Esther Taks, Priya A. Debisarun, Gaby Smits, Rob van Binnendijk, Fiona van der Klis, Lilly M. Verhagen, Marien I. de Jonge, Marc J.M. Bonten, Mihai G. Netea, Janneke H.H.M. van de Wijgert

SUPPLEMENTARY METHODS

The sample collection protocol, the antibody measurement procedure, and outcome definitions have been previously described in detail [19].

Inclusion and exclusion criteria

Participants were healthcare workers, including doctors, nurses, paramedics, and support staff. They were 18 or older and had to be in possession of a smartphone. Participants were also expected to be working in direct contact with SARS-CoV-2 infected patients. The primary exclusion criteria were known allergy to BCG, active or latent *Mycobacterium tuberculosis* infection (as judged by the local Principal Investigator in each hospital), any other active infection, immunocompromised state, malignancy or lymphoma in the past two years, current or planned pregnancy, any vaccination in the past 4 weeks, having a hospital employment contract of less than 22 hours per week, or expected work absence of at least 4 weeks.

Sample collection

About half of the participants were recruited in the three core hospitals (University Medical Center (UMC) Utrecht, Radboud UMC, and Leiden UMC) and invited to participate in sampling in their own hospital at about 3 months (M3; June 2020) and 12 months (M12; April 2021) after study vaccination. One serum aliquot per participant was transported to the Center for Immunology of Infections and Vaccines at the National Institute for Public Health and the Environment (RIVM in Dutch) in Bilthoven, the Netherlands, for antibody testing.

The remaining participants were recruited in six other hospitals (Noordwest Ziekenhuis Alkmaar, Haga Ziekenhuis Den Haag, Canisius-Wilhelmina Ziekenhuis Nijmegen, Sint Maartenskliniek Nijmegen, Jeroen Bosch Ziekenhuis Den Bosch, and Erasmus Medisch Centrum Rotterdam), and were invited to fingerprick sampling at home. Participants from core hospitals who could not attend an in-hospital sampling visit were also asked to conduct finger-prick sampling at home. The implementation of each at-home sampling round took several weeks; the first round took place from 9 Oct 2020 until 18 Dec 2020, and the second round from 14 April 2021 until 9 June 2021. The diary app was discontinued on 27 March 2021. We collected data on COVID-19-like symptoms, SARS-CoV-2 tests, and COVID-19 vaccinations between 27 March 2021 (diary app cessation) and the participant's final sampling date via Formdesk (Innovero Software Solutions BV, Wassenaar, Netherlands) and email.

Diary completeness

The app completion percentage was calculated for the period between vaccination and app discontinuation on 27 March 2021, and the total follow-up time as the number of days between vaccination and the M12 sampling date. The app completion percentage was calculated as the number of completed app entries (the number of days for which the participant completed the diary, regardless of whether the s/he formally withdrew from the study) divided by the expected number of app entries (the number of days between randomisation and diary app cessation on 27 March 2021). Participants were considered to have adequate follow-up data if the app completion

percentage was at least 80%.

Seroconversion window
The seroconversion window was defined as 14 days prior to the participant's M12 sampling date until the date of sampling. We used 14 days because previous studies have shown that the median systemic IgG seroconversion time is around 14 days for hospitalised patients [41], with more severe cases seroconverting at a faster rate than mild cases [1]. Furthermore, in mild cases, antibodies are reliably detected between 10-17 days after symptoms onset [1, 41, 42]. We conducted sensitivity analyses with seroconversion windows of 0 and 7 days instead of 14 days (Table S4). The numbers of participants that were excluded because they had an immune event within the seroconversion window are shown in the footnotes of Table S4.

Covariates

Work-related SARS-CoV-2 exposure risk
We combined several baseline workplace characteristics (job function and department, percent of work hours with direct patient contact, and planned work on a COVID-ward) into one workplace exposure risk variable, categorised as high, medium, or low risk as follows:

		Planned work in COVID-ward		
% work hours with patient contact		**Yes**	**Unknown**	**No**
	**0-25**	Medium	Medium	Low
	**26-50**	High	Medium	Low
	**51-75**	High	High	Medium
	**75+**	High	High	Medium

Positive SARS-CoV-2 infection
A positive SARS-CoV-2 infection was identified through self-reporting of a positive SARS-CoV-2 test of any type or seroconversion as described in the methods of the manuscript. Four participants reported a second infection during the one year follow-up. These participants were excluded from the total analysis population because the subgroup was too small to generate meaningful results.

Immune events, and vaccination types and doses
These covariates are described in the methods of the manuscript.

Overall infection severity and severity of individual symptoms
The following respiratory and non-respiratory symptoms could be reported in the diary app:

<u>Respiratory symptoms</u> (all reported on a scale of 0-5, with 0 meaning not present):
- Nose cold (*Neusverkouden*)
- Sore throat (*Keelpijn*)
- Cough (*Hoesten*)
- Dyspnea/shortness of breath (*Kortademig*)
- Loss of smell/taste (*Reuk en/of smaakverlies*)

<u>Non-respiratory symptoms</u> (all reported on a scale of 0-5 except fever):
- Fever (*Koorts*), defined as a temperature of 38 °C or above
- Cold shivers (*Koude rillingen*)
- Muscle pain (*Spierpijn*)

- Fatigue (*Vermoeidheid*)
- Headache (*Hoofdpijn*)
- Diarrhoea (*Diarree*)

We used the World Health Organization (WHO) definitions for infection severity [17], and further subcategorised WHO mild category into very mild and mild subcategories. The definitions of episode severity are described below. Chronic symptoms that were consistently reported by participant without a clear link to a test date or episode of respiratory symptoms were ignored.

- *WHO moderate:* Participant had clinical signs of pneumonia and hospitalisation due to SARS-CoV-2 but did not require high flow oxygen therapy.
- *WHO mild:* Participants experienced symptoms due to SARS-CoV-2 but were not hospitalised and did not have evidence of pneumonia or hypoxia.
  - *Subcategory mild:* Participant reported a fever (temperature 38+) for more than one week AND/OR dyspnoea reaching level 4 or 5 for more than one week AND/OR any other respiratory symptoms with at least one symptom other than loss of smell/taste reaching level 4 or 5 for more than one week AND/OR a total symptomatic episode lasted for more than 28 days.
  - *Subcategory very mild:* Participant reported symptoms during infection episode that did not reach the level of subcategory mild as described above.
- *WHO asymptomatic:* Participant did not experience any symptoms during relevant period, infection episode was detected via SARS-CoV-2 diagnostic testing or serology.

When it was impossible to draw conclusions about episode severity from the available data, the episode severity was coded as unknown.

Overall acute episode duration and duration of individual symptoms
The overall acute episode duration was calculated as the number of consecutive days during which a participant reported symptoms, excluding Long COVID or long-term loss of smell/taste. Similar definitions were used for each individual symptom. Long COVID (lingering symptoms other than standalone loss of smell/taste) and long-term loss of smell/taste were defined as continuing to report symptoms for at least 60 consecutive days after the end of the acute infection episode [43].

Regression model building
Associations between covariates and $\log_{10}$-transformed M12 anti-S1 and anti-N concentrations were assessed using univariable and multivariable linear regression models for the total analysis population (Table 3, Table S3A). First, all covariates were assessed in univariable models, and those with $p<0.05$ were considered for inclusion in multivariable models. However, we decided to exclude:
- work-related SARS-CoV-2 exposure risk because it determines someone's likelihood of becoming infected rather than the immune response to infection.
- past TB test results because the 'not done' and 'unknown' categories were the only categories that were statistically significant in the anti-S1 univariable model.
- having received an influenza vaccination in the winter of 2019/2020 because the 'missing' category was the only statistically significant category in the anti-S1 univariable model.
- history of other pulmonary disease (other than hay fever or asthma) because of low prevalence.

We added these variables to the final model in sensitivity analyses (Figure S6).

In addition, the use of anti-hypertensive medication was kept in the multivariable anti-N model despite it no longer being significant because it has been consistently associated with SARS-CoV-2 infection risk and severity. There may be a biological reason for this: patients with high blood

pressure have upregulated angiotensin-converting enzyme 2 (ACE2), which is the entry receptor for SARS-CoV-2 [44, 45].

Various covariates were strongly correlated. Severity of infection was strongly correlated with having an infection (a prerequisite) and with duration of acute infection episode (included in the severity definition). We therefore created a new variable called ‘overall infection severity’, including a ‘no infection’ category, and did not add acute episode duration to the final multivariable models (we did add it as a sensitivity analysis as shown in Figure S6). The severities and durations of individual symptoms were correlated with each other (see correlation matrix below) and with overall infection severity and were therefore only assessed in univariable models.

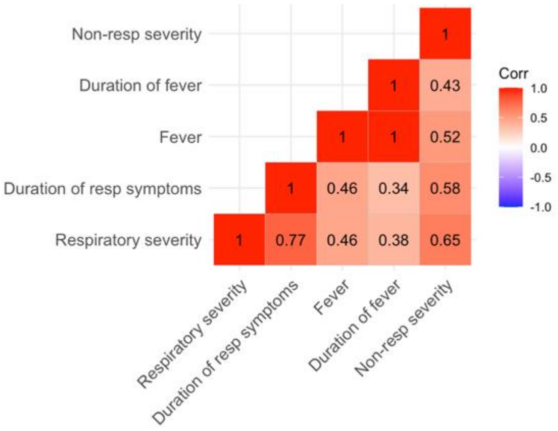

The COVID-19 vaccine type was correlated with the number of doses received due to vaccination recommendations and roll-out procedures: most healthcare workers qualified for mRNA vaccination early on during roll-out, whereas the vector vaccines were rolled out later and the primary schedule of vector vaccine Jcovden consisted of only one dose. Therefore, by the end of our follow-up period, most participants who received a mRNA vaccine reported having received two doses of that vaccine, whereas all participants that received a vector vaccine only reported one dose. We therefore created a new COVID-19 vaccination variable with the categories not vaccinated, mRNA vaccine 1 dose, mRNA vaccine 2 doses, and vector vaccine 1 dose.

In addition to the above-described univariable and multivariable analyses on the total analysis population, we also ran univariable models on a subgroup of participants who experienced an infection (Table S3B).

The final models adhered to the univariable and multivariable linear regression assumptions, and the variance inflation factor (VIF) values of the multivariable models were less than 5, indicating a lack of multicollinearity.

SUPPLEMENTARY RESULTS

Sensitivity analyses results

Sensitivity analyses adding more covariates to the final multivariable models produced similar results (Figure S6). Overall infection severity and overall acute episode duration could not be added due to multicollinearity.

Sensitivity analyses reducing the seroconversion windows from 14 to 7 or 0 days also produced similar results with two exceptions. The positive associations between M12 anti-S1 $\log_{10}$ concentration and mild or moderate infection severity (compared to no infection) increased when the seroconversion period was reduced, and having received one dose of a mRNA or vector vaccine

(compared to no vaccination) decreased, but estimates were still statistically significant (Table S4). We explored reasons for the changes in the strengths of the associations. Only two moderate infections were included in the total analysis population after applying a 14 day seroconversion window, and this increased to three infections when the window was reduced to 7 or 0 days. These numbers are too small to enable meaningful conclusions. The observed increase in the estimate for mild infections may be due to potential boosting by COVID-19 vaccination after experiencing an infection. Most of the participants in the 14-day seroconversion window were in that window because of a recent vaccination, not because of a recent infection.
