# Supplementary material for: Determinants of Systemic SARS-CoV-2-Specific Antibody Responses to Infection and to Vaccination: A Secondary Analysis of Randomised Controlled Trial Data
Source: Vaccines (Basel). 2024 Jun 20;12(6):691. doi: 10.3390/vaccines12060691 (PMC11209274; doi:10.3390/vaccines12060691)
Supplement: Supplementary file 1 [file vaccines-12-00691-s001.zip › vaccines-3040160 - Vaccines Supplementary Tables and Figures.pdf]

SUPPLEMENTARY TABLES AND FIGURES

Determinants of systemic SARS-CoV-2-specific antibody responses to infection and to vaccination: a secondary analysis of randomised controlled trial data

Authors: Juana Claus, Thijs ten Doesschate, Esther Taks, Priya A. Debisarun, Gaby Smits, Rob van Binnendijk, Fiona van der Klis, Lilly M. Verhagen, Marien I. de Jonge, Marc J.M. Bonten, Mihai G. Netea, Janneke H.H.M. van de Wijgert

Figure S1: Distribution of $\log_{10}$ concentrations of A) anti-S1 IgG and B) anti-N IgG at M12

A)

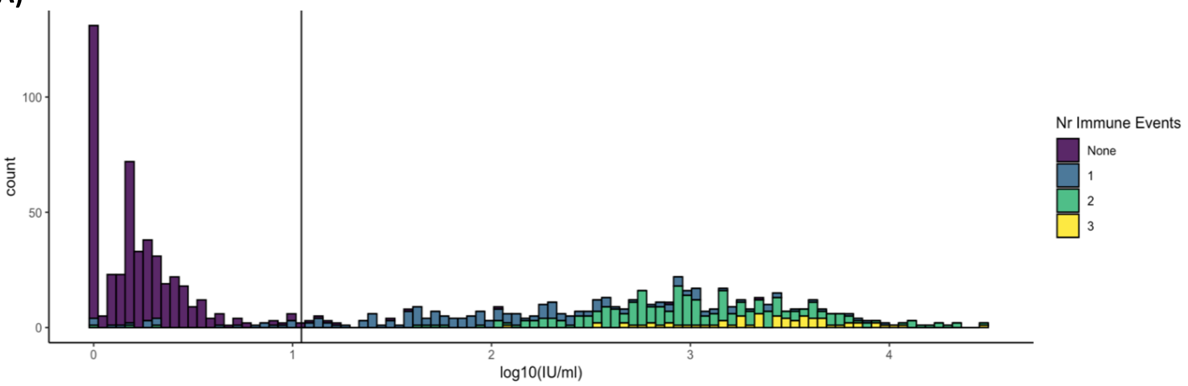

B)

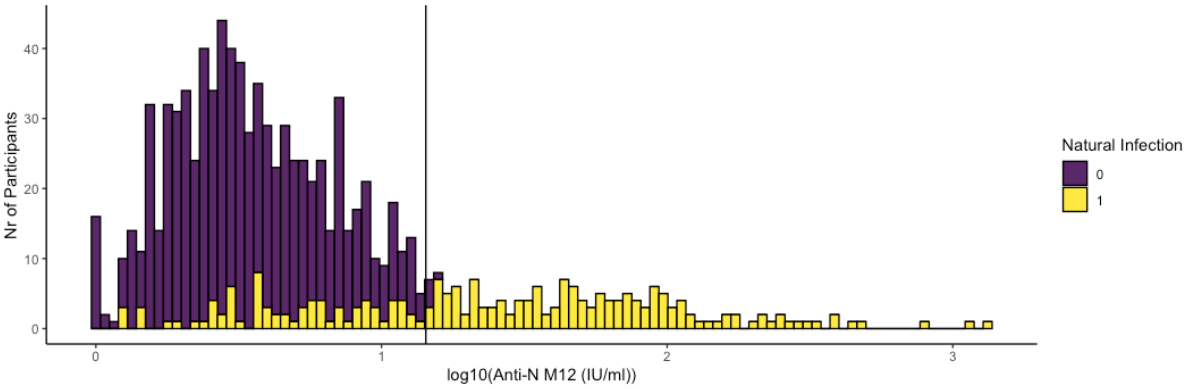

Systemic antibody concentrations were $\log_{10}$ transformed (after pseudocount of +1). The vertical lines in the panels marks the thresholds for anti-S1 and anti-N seropositivity, respectively. An immune event is either one SARS-CoV-2 infection or one dose of a COVID-19 vaccine.

Figure S2: Timelines of first COVID-19 vaccination (A-C) or SARS-CoV-2 infection (D-F) for seroconversion windows of 14, 7 or 0 days

A) First dose of COVID-19 vaccine: analysis population
seroconversion window = 14 days

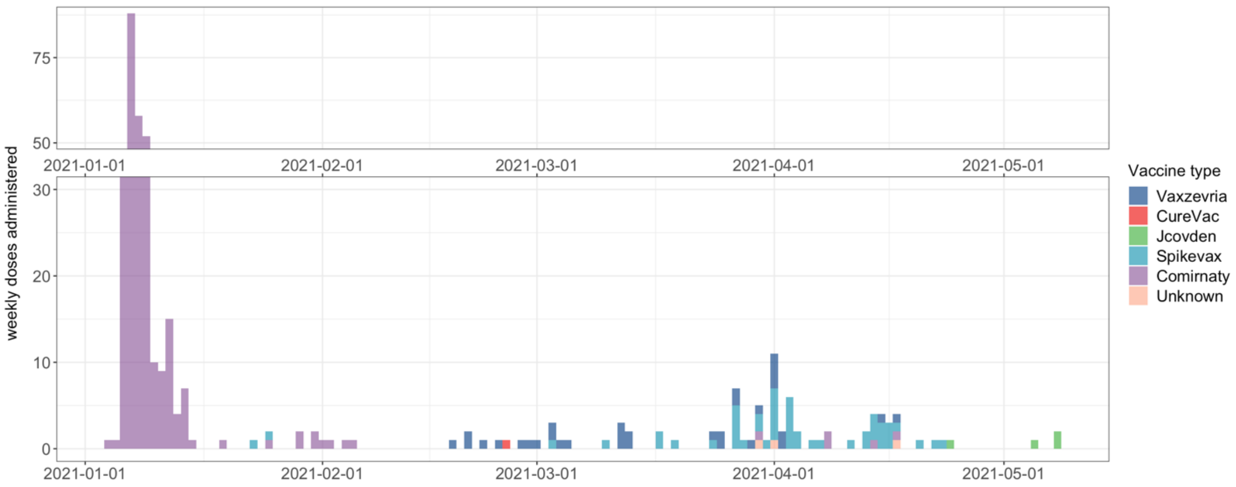

B) First dose of COVID vaccine: sensitivity analysis
seroconversion window = 7 days

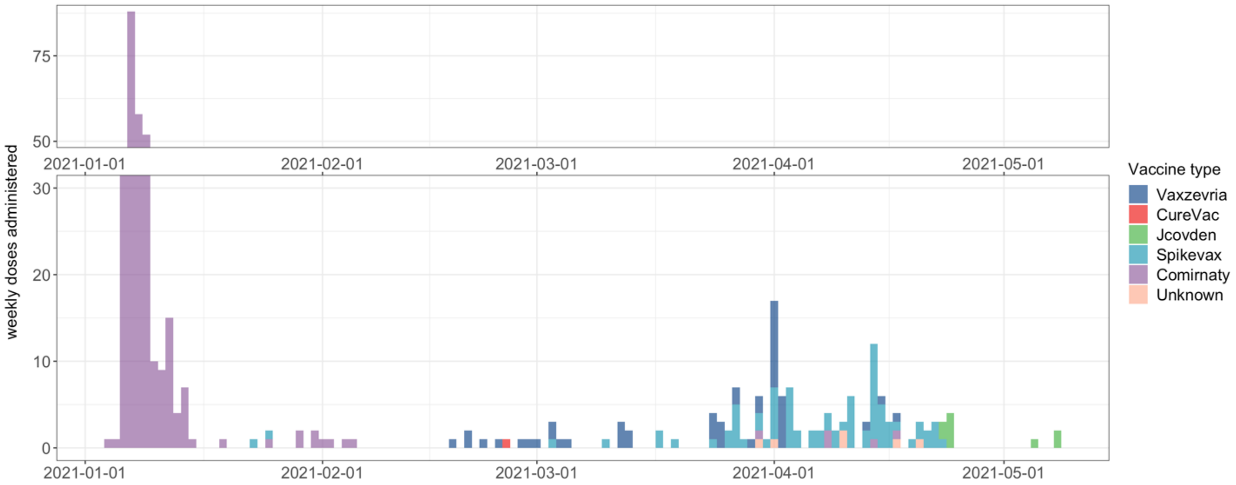

C) First dose of COVID-19 vaccine: sensitivity analysis
seroconversion window = 0 days

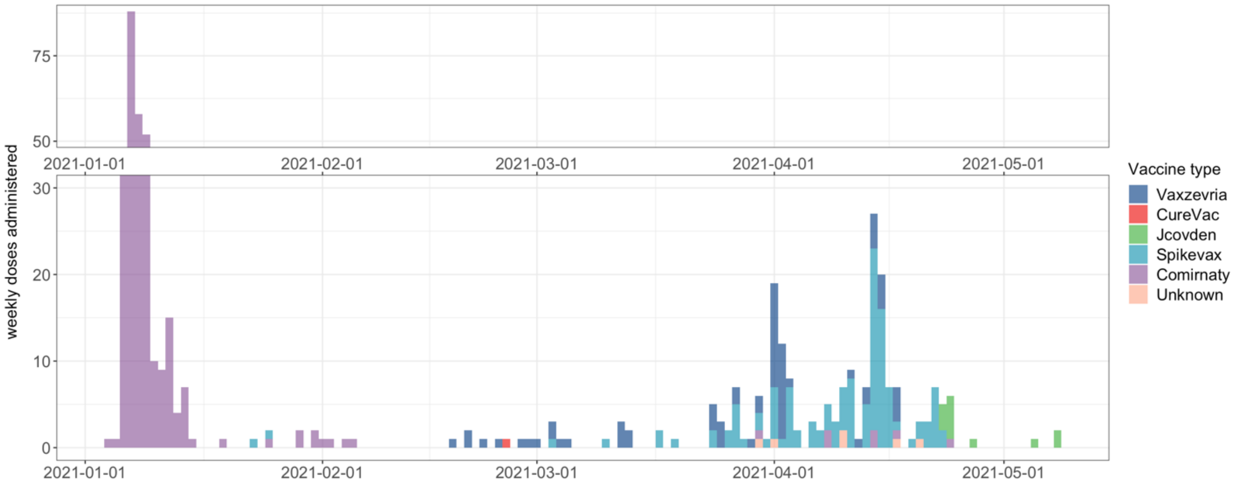

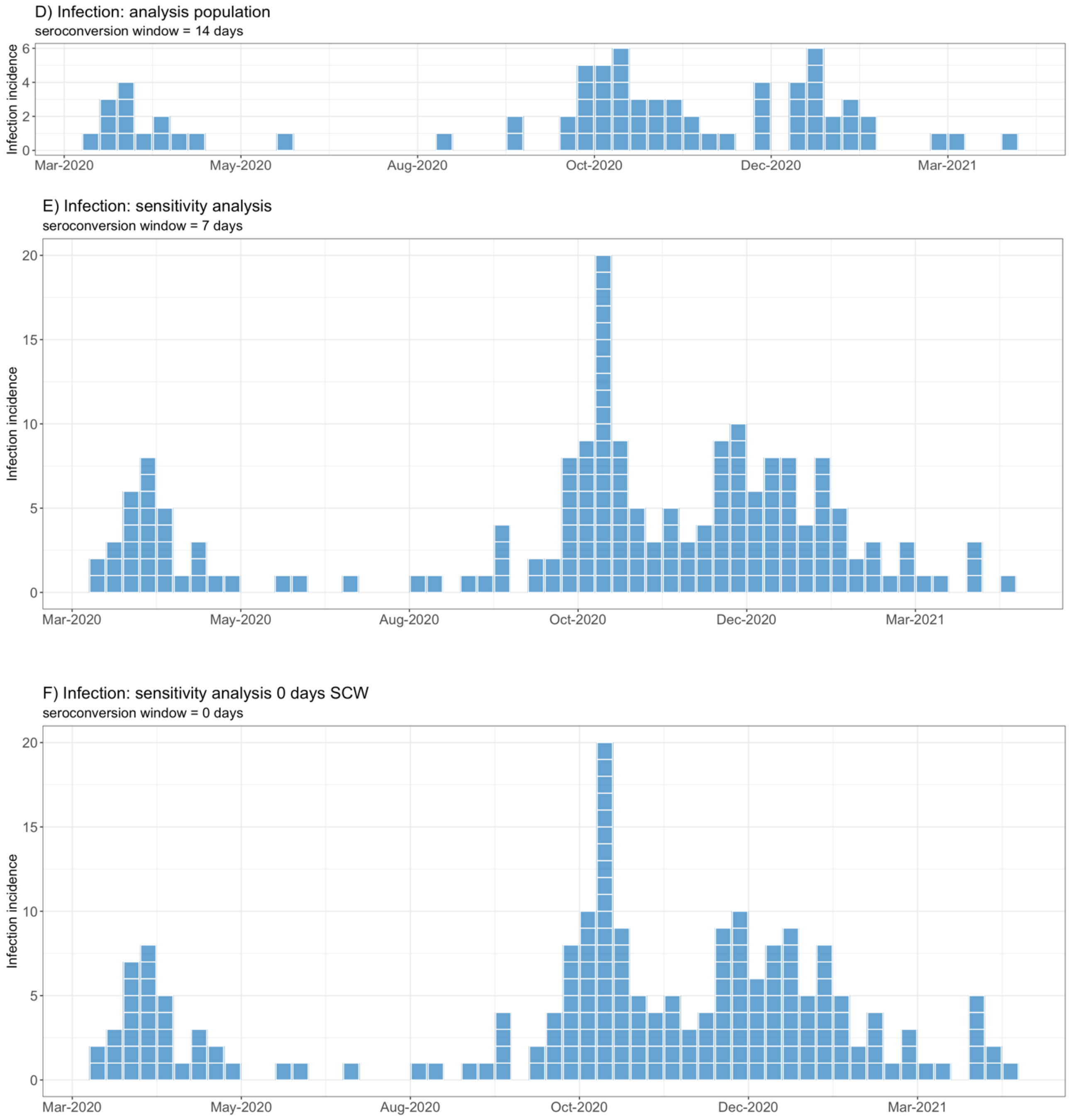

A+D. Window period is 14 days. The analysis population includes 970 participants and 223 infections. Four participants were in the seroconversion window for an infection and 125 for the first dose of a vaccine.
B+E. Window period is 7 days. The sensitivity analysis population includes 1,024 participants and 236 infections. Four participants were in the seroconversion window for an infection and 71 for the first dose of a vaccine.
C+F. Window period is 0 days. The sensitivity analysis population includes 1,099 participants and 249 infections.

Table S1: Participant baseline characteristics and immune events during follow-up with participants in 14-day seroconversion window removed from both populations

**A. Baseline characteristics**<br>Cells contain n (% of N) unless stated otherwise		**Analysis population**<br>**N= 970**	**Randomised population**<br>**N=1,370[1]**	**p[2,3]**
**Age in years, mean (SD)**		42.51 (12.59)	41.57 (12.63)	0.076
**Female**		724 (74.7)	1003 (73.2)	0.468
**Work-related exposure[4]**	Low	148 (15.3)	196 (62.3)	0.540
	Medium	240 (24.7)	321 (23.4)	
	High	582 (60.0)	853 (62.3)	
**Smoking status**	Current	65 (6.7)	113 (8.2)	0.222
	Former	302 (31.1)	393 (28.7)	
	Never	603 (62.2)	864 (63.1)	
**History of BCG vaccination**		174 (17.9)	234 (17.1)	0.629
**Past tuberculosis test results[5]**	Negative	640 (66.0)	922 (67.3)	0.766
	Positive (either or both)	94 (9.7)	124 (9.1)	
	Never tested	224 (23.1)	312 (22.8)	
	Unknown (both)	12 (1.2)	12 (0.9)	
**Respiratory infection in winter 2019-2020**				0.959
	No	713 (73.5)	1000 (73.0)	
	Yes, with fever	79 (8.1)	115 (8.4)	
	Yes, no fever	178 (18.3)	255 (18.6)	
**Influenza vaccination in winter 2020-2021[6]**				**<0.001**
	Yes	528 (54.4)	613 (44.7)	
	No	311 (32.1)	388 (28.3)	
	Missing	131 (13.5)	369 (26.9)	
**Influenza vaccination prior to follow-up**		560 (57.7)	798 (58.2)	0.836
**Any other vaccination in past year[7]**		105 (10.8)	154 (11.2)	0.803
**Current use of anti-hypertensive medication**		61 (6.3)	87 (6.4)	1.000
**History of cardiovascular disease**		23 (2.4)	32 (2.3)	1.000
**Current use of anti-diabetic medication**		5 (0.5)	7 (0.5)	1.000
**History of asthma**		69 (7.1)	95 (6.9)	0.932
**History of hay fever**		288 (29.7)	410 (29.9)	0.938
**History of other pulmonary disease**		23 (2.4)	30 (2.2)	0.881
**Any lung disease (previous three combined)**		329 (33.9)	462 (33.6)	0.957
**B. Immune events**<br>Cells contain n (% of N) unless stated otherwise		**Analysis population**<br>**N= 970**	**Randomised population**<br>**N=1,370[1]**	**p[2,3]**
**# Immune events[8]**	0	453 (46.7)	679 (50.7)	0.202
	1	187 (19.3)	223 (16.7)	
	2	260 (26.8)	351 (26.2)	
	3	70 (7.2)	86 (6.4)	
**Immune event type**	None	453 (46.7)	679 (49.6)	**<0.001**
	Infection[9]	123 (12.7)	152 (11.1)	
	Vaccine 1 dose	64 (6.6)	57 (5.2)	
	Vaccine 2 doses	230 (23.7)	320 (23.4)	
	Infection + 1 dose	30 (3.1)	30 (2.2)	
	Infection + 2 doses	70 (7.2)	86 (6.3)	
**COVID-19 vaccine product/dose[10]**	None	576 (59.4)	862 (62.9)	0.052
	mRNA 1 dose	55 (5.7)	58 (4.2)	
	mRNA 2 doses	300 (30.9)	399 (29.1)	
	Vector 1 dose	36 (3.7)	38 (2.8)	
	Vector 2 doses	0 (0.0)	7 (0.5)	
	Unknown 1 dose	3 (0.3)	6 (0.4)	
**Had SARS-CoV-2 infection during follow-up[11]**		223 (21.2)	268 (20.3)	0.094
**Infection severity[12]**	No infection	747 (77.0)	1102 (80.4)	0.527
	Asymptomatic	33 (3.4)	38 (2.8)	
	Very Mild	120 (12.4)	148 (10.8)	
	Mild	44 (4.5)	52 (3.8)	
	Moderate	2 (0.2)	2 (0.1)	
	Unknown	24 (2.5)	28 (2.0)	
**Acute duration of infection[13]**	0 days/no infection	782 (80.6)	1136 (82.9)	0.885
	0-1 weeks	29 (3.0)	40 (2.9)	
	1- 2 weeks	45 (4.6)	52 (3.8)	
	2-3 weeks	32 (3.3)	41 (3.0)	
	3-4 weeks	17 (1.8)	18 (1.3)	
	4+ weeks	15 (1.5)	16 (1.2)	
	Lingering	22 (2.3)	28 (2.0)	
	Ongoing/unknown	28 (2.9)	39 (2.8)	

**Long COVID**	No<br>Yes<br>Unknown	193 (86.5)<br>19 (8.5)<br>11 (4.9)	230 (85.8)<br>23 (8.6)<br>15 (5.6)	0.947
**Long term loss of smell/taste**	No<br>Yes<br>Unknown	211 (94.6)<br>6 (2.7)<br>6 (2.7)	252 (94.0)<br>8 (3.0)<br>8 (3.0)	0.962

Abbreviations: BCG= Bacillus Calmette-Guerin; M=Month; SD=standard deviation.

1. Participants that were in the 14-day seroconversion window for an infection (N=4) or the first dose of a COVID-19 vaccination (N=133) were removed from the randomised population. An additional 17 participants were in the 14-day seroconversion window for a second COVID-19 vaccination. They were recoded as having received only one dose.
2. Chi-squared tests for categorical variables and Wilcoxon rank sum test for continuous variables.
3. Statistical test comparing baseline characteristics between the analysis (N=970) and randomised populations (N=1,370).
4. Work related exposure is a combination of participants expected to work in a COVID-19 ward and the percentage of hours with direct patient contact (Supplementary Methods).
5. Tuberculosis tests include the Mantoux and/or TB QuantiFERON tests.
6. Only the missing category is statistically significantly different between the analysis and randomised populations.
7. Included DTaP-IPV, hepatitis A, hepatitis B, yellow fever, typhoid, rabies, mumps-measles-rubella, meningococcal, pneumococcal, *Haemophilus influenza* type B, Ebola, tick-borne encephalitis, human papillomavirus, and unknown.
8. An immune event is considered to be either one SARS-CoV-2 infection or one dose of a COVID-19 vaccine.
9. No-one in the analysis population, and four persons in the randomised population, had more than one infection (two each).
10. COVID-19 vaccines available in the Netherlands during the study period are listed in the methods. In addition, one person received an experimental mRNA vaccine by CureVac N.V. in a clinical trial setting. That vaccine was never marketed due to insufficient efficacy, but the person was included in the mRNA vaccines group.
11. A natural infection was defined as a reported positive test (by the participants through the diary app) or identified by serology (evidence of anti-S1 at sampling round 1 or both anti-S1 and anti-N at sampling round 2). None of the participants in the analysis population reported a positive PCR test at baseline.
12. Participants with an unsure infection status at M12 (never reported a positive test and/or seropositive for anti-N but not anti-S1 at M12; n=36) were considered as never having had an infection during follow-up in the randomised population and were removed from the analysis population.
13. Participants who never had an infection were included in the 0 days/no infection category.

Figure S3: Spearman correlations between M12 anti-S1 and anti-N log_{10} concentrations and time since SARS-CoV-2 infection (A-B) or time since first dose of a COVID-19 vaccine (C)

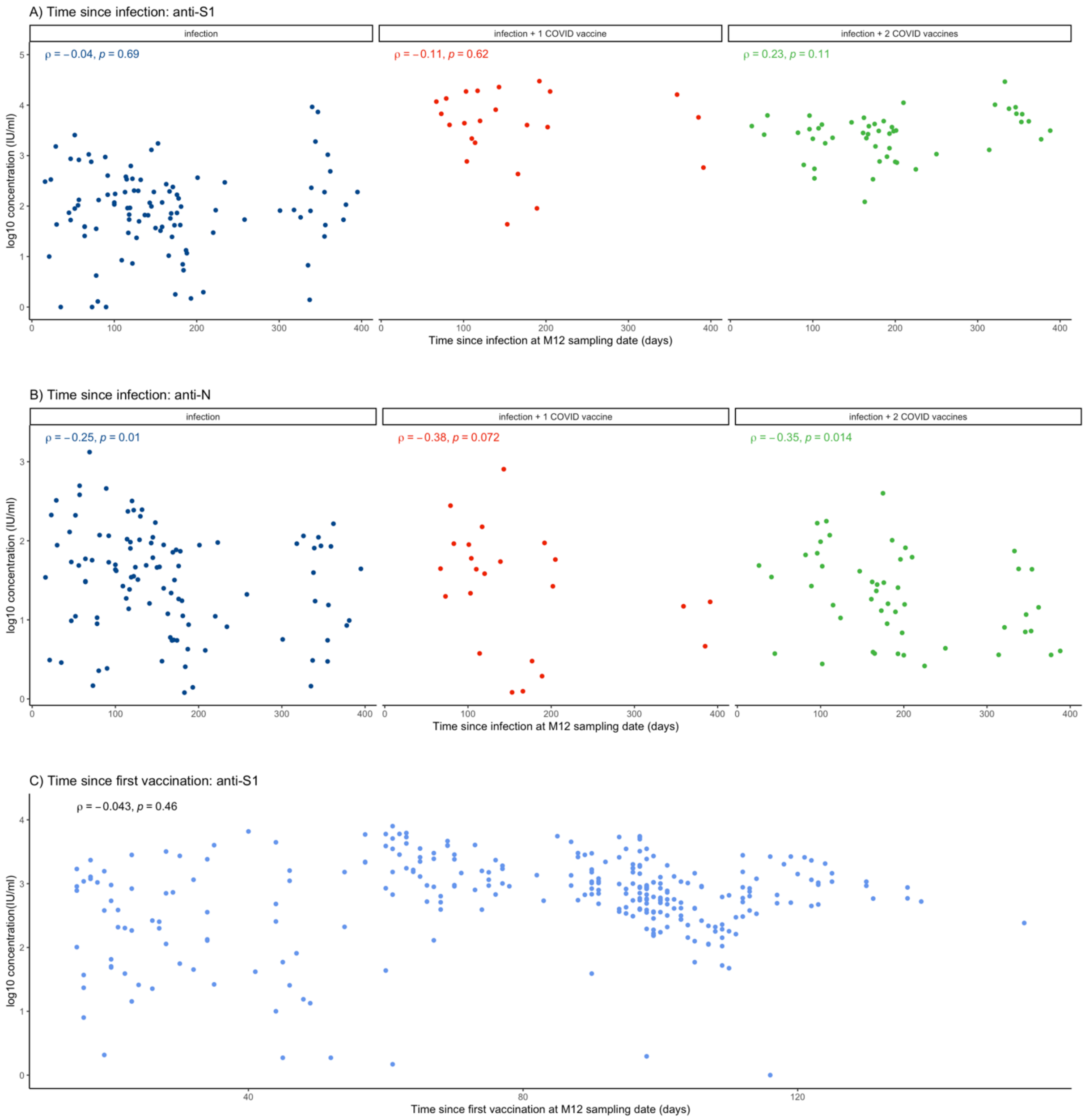

A-B. N=174; 49 participants with an unknown infection date excluded.
C. N=294; only includes participants who received at least one dose of a COVID-19 vaccine but never experienced a SARS-CoV-2 infection.

Table S2: Mean M12 anti-S1 log_{10} concentrations by COVID-19 vaccine dose and type

		M12 anti-S1		M12 anti-N	
**Vaccine type and dose[1]**	**N=747[2] (%)**	**Mean log_{10} concentration (SD) in IU/ml**	**Geometric mean (SD)[3] in IU/ml**	**Mean log_{10} concentration (SD) in IU/ml**	**Geometric mean (SD)[3] in IU/ml**
**None**	453 (60.6)	0.25 (0.32)	1.78 (2.09)	0.54 (0.28)	3.47 (1.91)
**Comirnaty 1**	4 (0.54)	2.08 (0.66)	120.23 (4.57)	0.44 (0.10)	2.75 (1.26)
**Comirnaty 2**	226 (30.3)	2.90 (0.54)	794.33 (3.47)	0.53 (0.26)	3.39 (1.82)
**Spikevax 1**	31 (4.1)	2.75 (0.75)	562.34 (5.62)	0.57 (0.29)	3.72 (1.95)
**Spikevax 2**	3 (0.40)	3.09 (0.32)	1230.23 (2.09)	0.63 (0.29)	4.27 (1.95)
**Vaxzevria 1**	25 (3.4)	1.78 (0.73)	60.26 (5.37)	0.51 (0.23)	3.24 (1.70)
**Jcovden 1**	3 (0.40)	1.93 (0.25)	85.11 (1.78)	0.55 (0.34)	3.55 (2.19)
**CureVac 2**	1 (0.13)	2.11 (NA)	128.82 (NA)	0.65 (NA)	4.47 (NA)
**Unknown 1**	1 (0.13)	0.32 (NA)	2.09 (NA)	0.24 (NA)	1.74 (NA)

Abbreviations: IU/ml= international unit per millilitre; M=month, SD=standard deviation.

1. In the Netherlands, the COVID-19 vaccines available during the study period were Spikevax (Moderna Biotech, Cambridge, MA, USA), Comirnaty (Pfizer/BioNTech, New York, NY, USA), Vaxzevria (AstraZeneca AB, Sodertalje, Sweden), and Jcovden (Janssen Vaccines, Leiden, Netherlands). In addition, one participant received an experimental vaccine by CureVac N.V. in a clinical trial setting. This vaccine was never marketed due to insufficient efficacy.
2. N=747; participants who experienced an infection only were excluded (n=223).
3. Geometric means are the antilog 10^x of the log_{10} concentrations.

Figure S4: Mean M12 anti-S1 and anti-N log_{10} concentrations for infection episode duration and individual symptom severity and duration

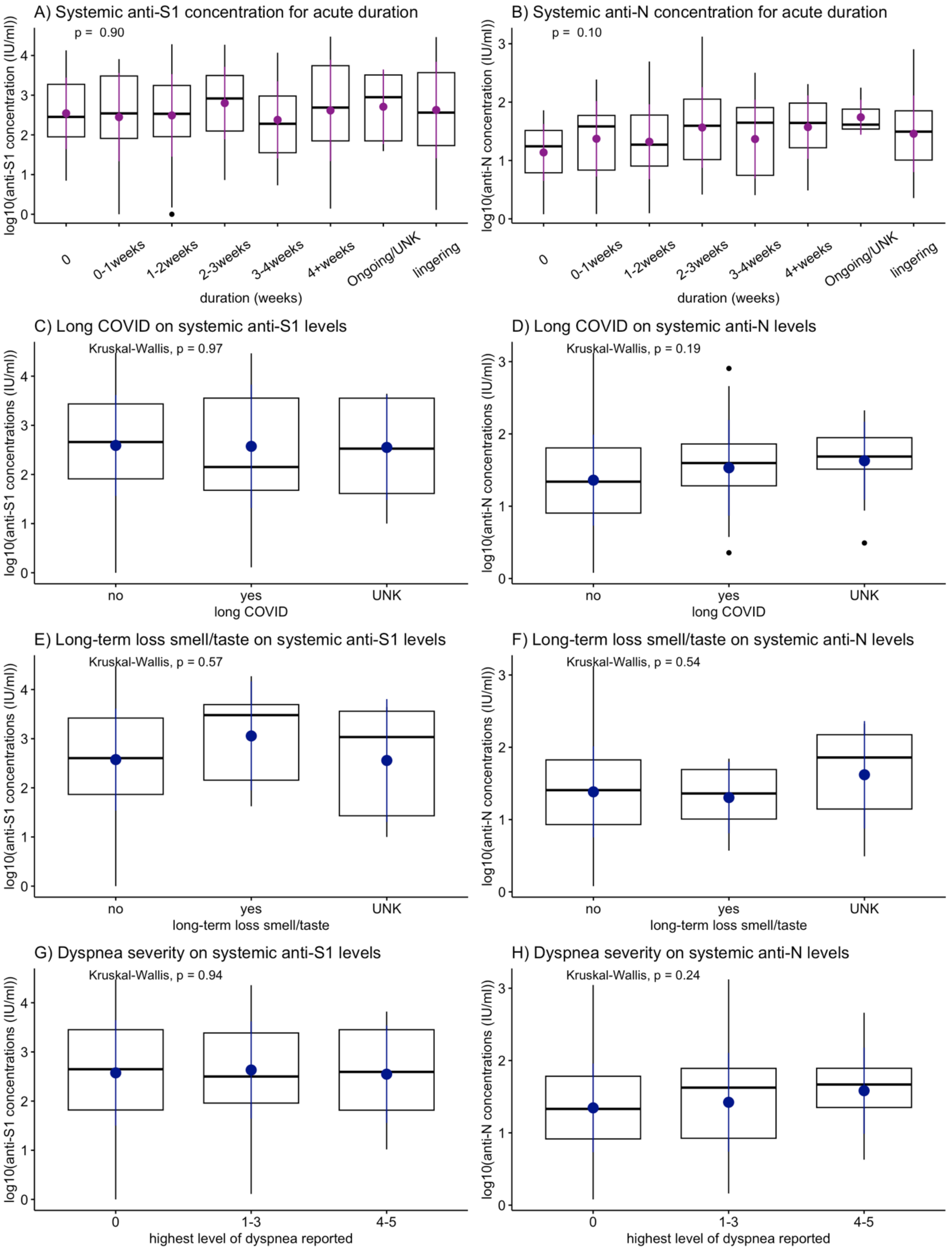

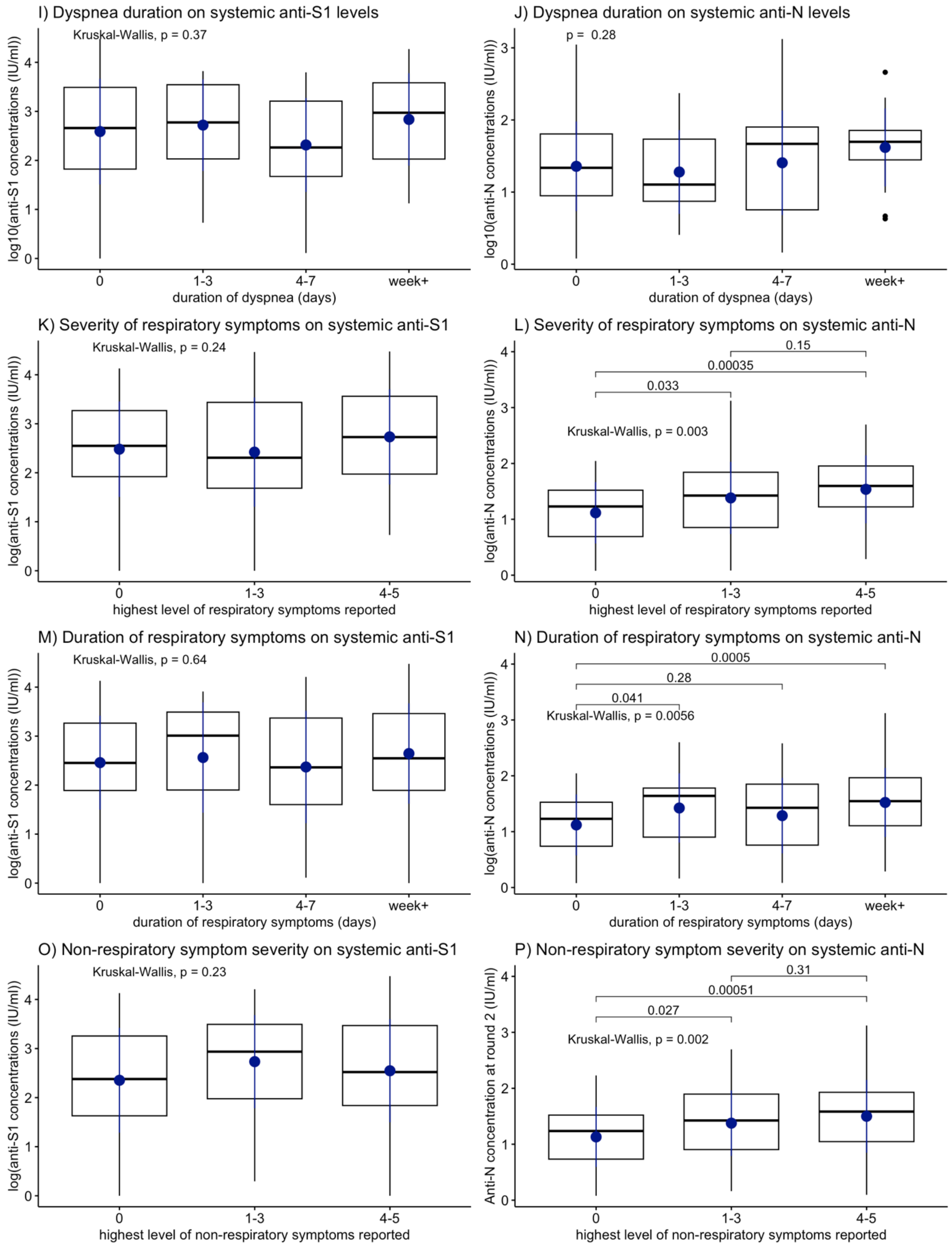
I) Dyspnea duration on systemic anti-S1 levels
Kruskal-Wallis, p = 0.37
log10(anti-S1 concentrations (IU/ml))
0
1-3
4-7
week+
duration of dyspnea (days)
J) Dyspnea duration on systemic anti-N levels
p = 0.28
log10(anti-N concentrations (IU/ml))
duration of dyspnea (days)
K) Severity of respiratory symptoms on systemic anti-S1
Kruskal-Wallis, p = 0.24
log(anti-S1 concentrations (IU/ml))
4-5
highest level of respiratory symptoms reported
L) Severity of respiratory symptoms on systemic anti-N
0.15
0.00035
0.033
Kruskal-Wallis, p = 0.003
log(anti-N concentrations (IU/ml))
highest level of respiratory symptoms reported
M) Duration of respiratory symptoms on systemic anti-S1
Kruskal-Wallis, p = 0.64
log(anti-S1 concentrations (IU/ml))
duration of respiratory symptoms (days)
N) Duration of respiratory symptoms on systemic anti-N
0.0005
0.28
0.041
Kruskal-Wallis, p = 0.0056
log(anti-N concentrations (IU/ml))
duration of respiratory symptoms (days)
O) Non-respiratory symptom severity on systemic anti-S1
Kruskal-Wallis, p = 0.23
log(anti-S1 concentrations (IU/ml))
highest level of non-respiratory symptoms reported
P) Non-respiratory symptom severity on systemic anti-N
0.31
0.00051
0.027
Kruskal-Wallis, p = 0.002
Anti-N concentration at round 2 (IU/ml)
highest level of non-respiratory symptoms reported

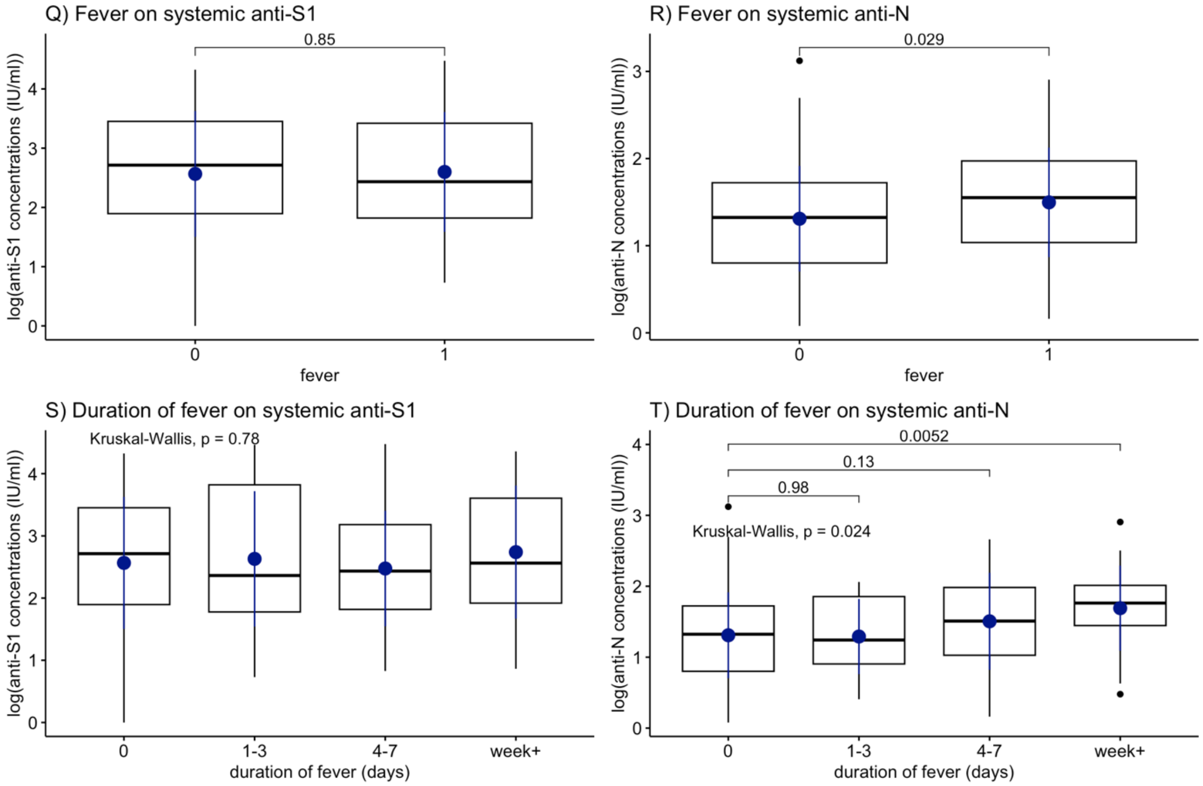

Systemic antibody concentrations were $\log_{10}$ transformed (after adding a pseudocount of 1).

A-B. Acute duration is defined as the days between the start of symptoms and end of symptoms surrounding an infection, excluding lingering non-respiratory symptoms. All infections that had an uncertain date (because the infection was identified through serology) and all ongoing infections at the end of follow-up were classified into the ongoing/unknown group. All infections with lingering symptoms, such as Long COVID or long-term loss of smell/taste, were classified as lingering.
C-F. Long COVID was defined as continuing to report symptoms other than standalone loss of smell/taste for at least 60 days after the end of the acute infection episode. Long-term loss of taste/smell was defined separately from Long COVID, as continuing to report standalone loss of smell/taste for at least 60 days after the end of the acute infection episode. Participants who were actively reporting symptoms past the end of their acute episode duration when the app was terminated, but had not yet reached two months were coded as unknown.
G-P. Severity was reported on a scale of 0-5, 0 indicating not present and 1-5 in increasing severity. Duration of symptoms is the number of days that the particular symptom was reported within the acute episode duration.
Q-T. Fever was defined as temperatures above 38°C.

Figure S5: Spearman correlations between M12 anti-S1 and anti-N log_{10} concentrations in participants who had a SARS-CoV-2 infection plus no, one or two vaccinations

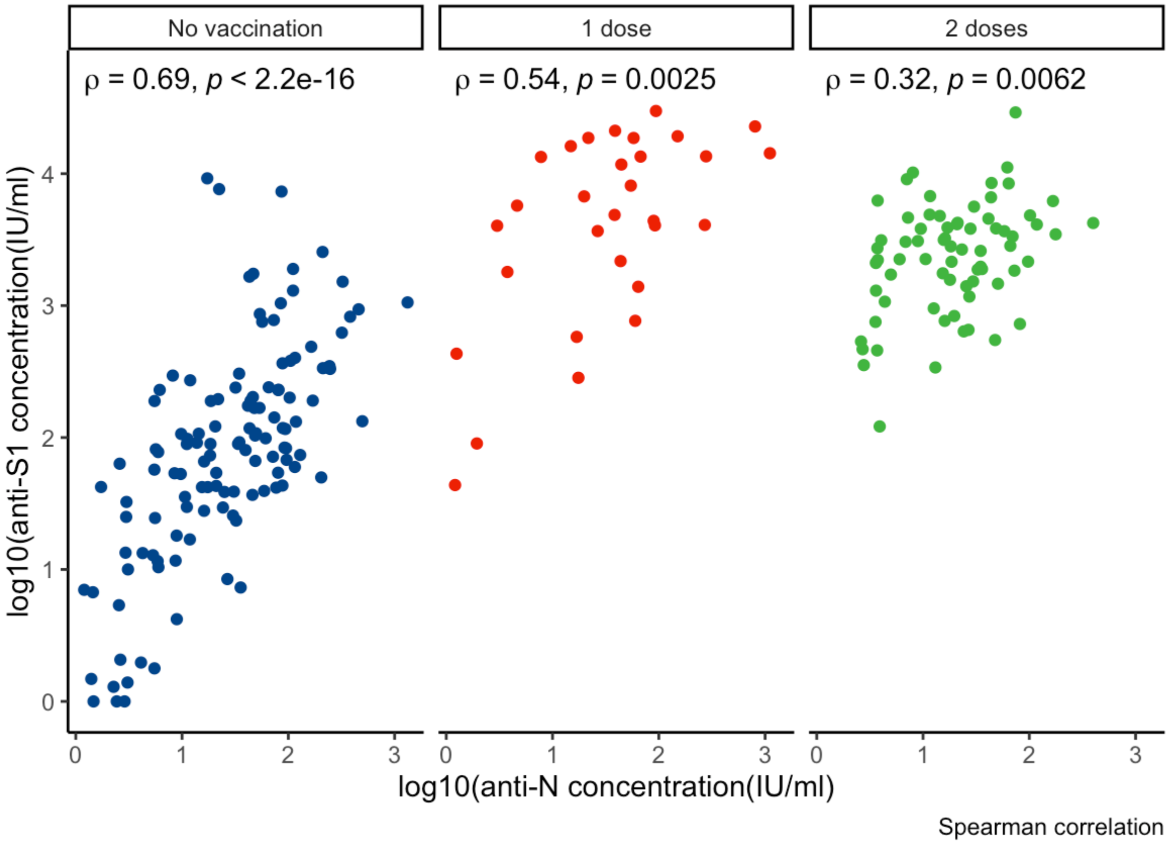

Systemic antibody concentrations were log_{10} transformed (after adding a pseudocount of 1).

Table S3: Univariable linear regression results for the full analysis population (A) and participants who experienced an infection (B)

**A. Analysis population[1]**		**M12 anti-S1 log_{10} concentration**				**M12 anti-N log_{10} concentration**			
		**Intercept (95% CI)**	**Model estimate (95% CI)**	**p**	**R^2**	**Intercept (95% CI)**	**Model estimate (95% CI)**	**p**	**R^2**
**Participant characteristics**									
Age (in years)		1.73 (1.43, 2.04)	-0.004 (-0.01, 0.003)	0.216	0.00	0.74 (0.63, 0.86)	-0.002 (-0.003, 0.002)	0.861	0.00
Male sex		1.52 (1.42, 1.62)	0.11 (-0.09, 0.31)	0.271	0.00	0.73 (0.69, 0.77)	0.02 (-0.06, 0.09)	0.625	0.00
Smoking		1.56 (1.45, 1.67)			0.00	0.73 (0.69, 0.78)			0.00
	Never		-0.04 (-0.40, 0.31)	0.816			-0.07 (-0.21, 0.06)	0.274	
	Current		-0.03 (-0.22, 0.16)	0.771			0.01 (-0.05, 0.09)	0.722	
	Former								
Work-related exposure[2]	Low	0.67 (0.46, 0.87)			0.15	0.67 (0.58, 0.75)			0.01
	Medium		0.39 (0.13,0.65)	**0.004**			0.03 (-0.07, 0.14)	0.551	
	High		1.30 (1.07, 1.53)	**<0.001**			0.10 (0.00, 0.10)	**0.049**	
Additional household members		1.55 (1.40, 1.70)	-0.002 (-0.07, 0.06)	0.956	0.00	0.72 (0.66, 0.77)	0.01 (-0.01, 0.03)	0.453	0.00
History of BCG vaccination		1.54 (1.44, 1.63)	0.06 (-0.17, 0.28)	0.623	0.00	0.72 (0.68, 0.76)	0.08 (-0.01, 0.16)	0.082	0.00
Past TB test results[3]	Negative	1.60 (1.50, 1.71)			0.01	0.72 (0.68, 0.76)			0.00
	Positive		0.12 (-0.18, 0.42)	0.447			0.06 (-0.05, 0.18)	0.284	
	Not done		-0.25 (-0.46, -0.04)	**0.020**			0.02 (-0.06, 0.10)	0.557	
	Unknown		-0.86 (-1.65, -0.07)	**0.033**			0.11 (-0.19, 0.41)	0.466	
Respiratory infection in winter 2019-2020[4]		1.55 (1.45, 1.66)			0.00	0.74 (0.70, 0.78)			0.00
	No								
	Yes, with fever		-0.17 (-0.49, 0.15)	0.299			-0.02 (-0.14, 0.10)	0.719	
	Yes, no fever		0.03 (-0.20, 0.26)	0.785			-0.04 (-0.13, 0.04)	0.344	
Influenza vaccine in winter 2019-2020		1.57 (1.41, 1.72)			0.01	0.75 (0.69, 0.81)			0.00
	No								
	Yes		-0.13 (-0.32, 0.07)	0.196			-0.04; (-0.12, 0.03)	0.242	
	Missing		0.37 (0.09, 0.65)	**0.010**			0.03; (-0.08, 0.14)	0.594	
Influenza vaccine prior to follow-up		1.61 (1.47, 1.74)	-0.10 (-0.28, 0.08)	0.260	0.00	0.73 (0.68, 0.78)	-0.002 (-0.07, 0.07)	0.995	0.00
Any other vaccination in past year[5]		1.56 (1.47, 1.66)	-0.16 (-0.45, 0.12)	0.251	0.00	0.74 (0.70, 0.77)	-0.05 (-0.16, 0.05)	0.333	0.00
Current use of anti-hypertensive medication		1.55 (1.46, 1.64)	-0.05 (-0.41, 0.31)	0.767	0.00	0.72 (0.69, 0.75)	0.20 (0.07, 0.39)	**0.003**	0.00
Current use of anti-diabetic medication		1.55 (1.46, 1.63)	0.10 (-1.12, 1.32)	0.871	0.00	0.73 (0.70, 0.77)	-0.27 (-0.73, 0.19)	0.249	0.00
History of cardiovascular disease		1.54 (1.45, 1.63)	0.22 (-0.36, 0.79)	0.463	0.00	0.73 (0.69, 0.76)	0.19 (-0.02, 0.41)	0.079	0.00
History of asthma		1.54 (1.45, 1.63)	0.09 (-0.25, 0.43)	0.585	0.00	0.73 (0.70, 0.77)	-0.01 (-0.14, 0.12)	0.912	0.00
History of hay fever		1.57 (1.47, 1.68)	-0.09 (-0.28, 0.10)	0.336	0.00	0.73 (0.69, 0.77)	0.01 (-0.06, 0.09)	0.701	0.00
History of other pulmonary disease		1.54 (1.45, 1.63)	0.32 (-0.25, 0.90)	0.273	0.00	0.73 (0.69, 0.76)	0.24 (0.02, 0.46)	**0.031**	0.00
Any lung disease (previous three combined)		1.56 (1.45, 1.67)	-0.04 (-0.23, 0.14)	0.666	0.00	0.73 (0.69, 0.77)	0.02 (-0.05, 0.09)	0.667	0.00
Intervention: BCG		1.59 (1.46, 1.71)	-0.08 (-0.26, 0.09)	0.355	0.00	0.75 (0.71, 0.80)	-0.04 (-0.11, 0.03)	0.235	0.00
**Immune events**									
Had a SARS-CoV-2 infection during follow-up		1.24 (1.14, 1.33)	1.35 (1.16, 1.54)	**<0.001**	0.17	0.54 (0.51, 0.56)	0.85 (0.79, 0.91)	**<0.001**	0.47
Acute infection duration[6]	0 days/no infection	1.29 (1.20, 1.38)			0.14	0.56 (0.54, 0.59)			0.44
	0-1 weeks		1.15 (0.67, 1.63)	**<0.001**			0.81 (0.66, 0.95)	**<0.001**	
	1- 2 weeks		1.20 (0.81, 1.58)	**<0.001**			0.76 (0.64, 0.88)	**<0.001**	

	2-3 weeks		1.51 (1.05, 1.96)	**<0.001**			1.00 (0.86, 1.14)	**<0.001**	
	3-4 weeks		1.08 (0.46, 1.71)	**0.001**			0.80 (0.61, 0.99)	**<0.001**	
	4+ weeks		1.32 (0.66, 1.98)	**<0.001**			1.01 (0.81, 1.21)	**<0.001**	
	Lingering		1.33 (0.79, 1.88)	**<0.001**			0.89 (0.73, 1.06)	**<0.001**	
	Ongoing/unknown		1.50 (1.02, 1.99)	**<0.001**			0.93 (0.78, 1.08)	**<0.001**	
Number of immune events[7]	0	0.25; (0.20, 0.30)			0.84	0.54; (0.50, 0.58)			0.25
	1		1.77 (1.68, 1.86)	**<0.001**			0.58 (0.50, 0.66)	**<0.001**	
	2		2.74 (2.65, 2.82)	**<0.001**			0.11 (0.05, 0.18)	**0.002**	
	3		3.12 (2.98, 3.25)	**<0.001**			0.74 (0.63.0.86)	**<0.001**	
Immune event type	None	0.25; (0.20, 0.30)			0.85	0.54; (0.50, 0.57)			0.47
	Infection		1.65 (1.55, 1.76)	**<0.001**			0.88 (0.81, 0.96)	**<0.001**	
	Vaccine 1 dose		2.00 (1.87, 2.14)	**<0.001**			-0.008 (-0.11, 0.09)	0.880	
	Vaccine 2 doses		2.66 (2.57, 2.74)	**<0.001**			-0.002 (-0.06, 0.06)	0.943	
	Infection+1 dose		3.37 (3.16, 3.55)	**<0.001**			0.96 (0.82, 1.10)	**<0.001**	
	Infection+2 doses		3.12 (2.98, 3.25)	**<0.001**			0.74 (0.64, 0.84)	**<0.001**	
Vaccine product and dose[8]	None	0.60; (0.54, 0.66)			0.70	0.72; (0.68, 0.77)			0.00
	mRNA 1 dose		2.49 (2.28, 2.70)	**<0.001**			0.15 (0.004, 0.29)	**0.044**	
	mRNA 2 doses		2.41 (2.30, 2.52)	**<0.001**			-0.02 (-0.09, 0.06)	0.639	
	Vector 1		1.46 (1.20, 1.72)	**<0.001**			0.03 (-0.14, 0.21)	0.728	
	Unknown 1 dose		2.14 (1.28, 3.01)	**<0.001**			0.44 (-0.15, 1.04)	0.140	
Infection severity	No infection	1.24 (1.14, 1.33)			0.17	0.54; (0.51, 0.56)			0.49
	Asymptomatic		1.31 (0.87, 1.75)	**<0.001**			0.60 (0.47, 0.73)	**<0.001**	
	Very Mild		1.28 (1.04, 1.53)	**<0.001**			0.82 (0.75, 0.89)	**<0.001**	
	Mild		1.44 (1.05, 1.82)	**<0.001**			1.11 (0.99, 1.23)	**<0.001**	
	Moderate		2.13 (0.37, 3.89)	**0.018**			1.13 (0.60, 1.65)	**<0.001**	
	Unknown		1.54 (1.03, 2.06)	**<0.001**			0.85 (0.70, 1.00)	**<0.001**	

Abbreviations: CI= confidence interval; M=month.

1. N=970. Linear regression with log_{10} transformed concentrations of systemic M12 anti-S1 and anti-N (after a pseudo-count of 1 was added) as outcome. Each covariate was individually considered in a univariable linear regression. For categorical variables, indicator variables were first created, as to not assume a linear relation between the categories.
2. Work related exposure is a combination of participants expected to work in a COVID ward and the percentage of hours with direct patient contact.
3. Tuberculosis tests include the Mantoux and/or TB QuantiFERON tests.
4. No respiratory tract infection is the reference.
5. The following other vaccinations were reported: DTaP-IPV, hepatitis A, hepatitis B, yellow fever, typhoid, rabies, mumps-measles-rubella, meningococcal, pneumococcal, *Haemophilus influenza* type B, Ebola, tick-borne encephalitis, human papillomavirus, and unknown.
6. Participants who never had a SARS-CoV-2 infection are included in the 0 days/no infection category.
7. An immune event is considered to be either one SARS-CoV-2 infection or one dose of a COVID-19 vaccine.
8. COVID-19 vaccines available in the Netherlands during the study period are listed in the methods. In addition, one person received an experimental mRNA vaccine by CureVac N.V. in a clinical trial setting. That vaccine was never marketed due to insufficient efficacy, but the person was included in the mRNA vaccines group. One mRNA vaccine dose was significantly associated with M12 anti-N log_{10} concentration, but this is due to the fact that a greater proportion of participants in that group experienced an infection (36.4%, compared 23.3% and 22.2% in the groups who received two mRNA vaccine doses or one vector vaccine dose, respectively).

B. Participants with a SARS-CoV-2 infection[1]		M12 anti-S1 log_{10} concentration				M12 anti-N log_{10} concentration			
		Intercept (95% CI)	Model estimate (95% CI)	p	R^2	Intercept (95% CI)	Model estimate (95% CI)	p	R^2
Overall severity[2]	Asymptomatic	2.54 (2.19, 2.90)			0.00	1.14 (0.93, 1.35)			0.07
	Very Mild		-0.03 (-0.43, 0.38)	0.898			0.22 (-0.02, 0.45)	0.070	
	Mild		0.13 (-0.34, 0.60)	0.587			0.51 (0.24, 0.79)	**<0.001**	
	Moderate		0.82 (-0.66, 2.31)	0.277			0.53 (-0.35, 1.40)	0.238	
Long COVID[3,4]	No	2.59 (2.44, 2.74)			0.00	1.36 (1.27, 1.45)			0.01
	Yes		-0.02 (-0.51, 0.48)	0.943			0.17; (-0.11, 0.65)	0.257	
	Ongoing/unknown		-0.04 (-0.68, 0.60)	0.901			0.27; (-0.13, 0.47)	0.168	
Long term loss of smell/taste[3,5]	Yes	2.57 (2.43, 2.72)			0.01	1.38 (1.30, 1.47)			0.00
	No		0.48 (-0.87, 0.84)	0.268			0.24 (-0.28, 0.75)	0.762	
	Ongoing/unknown		-0.02 (-0.37, 1.33)	0.967			-0.08 (-0.59, 0.44)	0.366	
Acute infection duration[3,6]	0 days	2.52 (2.18, 2.88)			0.02	1.13; (0.92, 1.34)			0.05
	0-1weeks		-0.08 (-0.60, 0.44)	0.758			0.24 (-0.07, 0.55)	0.122	
	1-2weeks		-0.04 (-0.50, 0.43)	0.873			0.19 (-0.08, 0.47)	0.172	
	2-3weeks		0.27 (-0.23, 0.78)	0.290			0.44 (0.14, 0.74)	**0.005**	
	3-4weeks		-0.15 (-0.76, 0.46)	0.629			0.24 (-0.12, 0.60)	0.197	
	4+weeks		0.09 (-0.55, 0.73)	0.791			0.44 (0.06, 0.82)	**0.022**	
	Lingering		0.10 (-0.45, 0.66)	0.730			0.33 (-0.01, 0.66)	0.054	
	Ongoing/unknown		0.27 (-0.26, 0.79)	0.314			0.36 (0.05, 0.67)	**0.023**	
Dyspnea severity[7,8]	0	2.57 (2.41, 2.74)			0.00	1.35 (1.25, 1.44)			0.01
	1-3		0.06 (-0.29, 0.41)	0.744			0.08 (-0.13, 0.29)	0.470	
	4-5		-0.03 (-0.57, 0.52)	0.923			0.23 (-0.09, 0.56)	0.150	
Duration of dyspnea[7] (days, continuous)		2.55 (2.41, 2.70)	0.01 (-0.01, 0.03)	0.233	0.01	1.36 (1.27, 1.44)	0.01 (-0.002, 0.02)	0.113	0.01
Respiratory symptoms severity[9,10]	0	2.48 (2.17, 2.79)			0.02	1.12; (0.93, 1.30)			0.06
	1-3		-0.07 (-0.44, 0.32)	0.757			0.27 (0.04, 0.49)	**0.019**	
	4-5		0.25 (-0.15, 0.65)	0.217			0.42 (0.18, 0.66)	**0.001**	
Respiratory symptoms duration[9,11] (days)		2.52 (2.32, 2.72)	0.002 (-0.01, 0.02)	0.743	0.00	1.26 (1.14, 1.38)	0.01 (0.004, 0.02)	**0.005**	0.04
Fever (yes/no)[12]		2.57 (2.40, 2.74)	0.03 (-0.26, 0.33)	0.824	0.00	1.31 (1.21, 1.41)	0.19 (0.01, 0.36)	**0.035**	0.02
Fever duration[12] (days)		2.57 (2.41, 2.73)	0.003 (-0.03, 0.03)	0.876	0.00	1.31 (1.22, 1.40)	0.03 (0.01, 0.05)	**0.002**	0.04
Non-respiratory symptoms severity[13,14]	0	2.35; (2.07, 2.64)			0.02	1.13 (0.96, 1.30)			0.06
	1-3		0.38 (-0.03, 0.79)	0.070			0.25 (0.003, 0.49)	**0.048**	
	4-5		0.19 (-0.15, 0.54)	0.271			0.37 (0.16, 0.58)	**<0.001**	

Abbreviations: CI=confidence interval; M=month.

1. Only those who experienced a SARS-CoV-2 infection were included. Linear regression with log_{10} transformed concentrations of systemic M12 anti-S1 and anti-N (after a pseudo-count of 1 was added) as outcome. Each covariate was individually considered in a univariable linear regression and categorical variables were considered ordinal.
2. N=199: Includes only participants who experienced a SARS-CoV-2 infection and an additional 24 participants were removed due to unknown infection severity. Includes only two moderate infections.
3. N=223: Includes only participants who experienced a SARS-CoV-2 infection.
4. Long COVID was defined as continuing to report symptoms other than standalone loss of smell/taste for at least 60 days after the end of the acute infection episode.
5. Long-term loss of taste/smell was defined as continuing to report standalone loss of smell/taste for at least 60 days after the end of the acute infection episode.
6. Acute duration of zero is the reference. Acute duration is defined as the days between the start of symptoms and end of symptoms surrounding an infection, excluding lingering non-respiratory symptoms. All infections that had an uncertain date (because the infection was identified through serology only) and all ongoing infections at the end of follow-up were classified into the unknown/ongoing group. All infections with lingering symptoms, such as Long COVID or long-term loss of smell/taste were classified into the lingering category.

7. N=218: Includes only participants who experienced a SARS-CoV-2 infection and an additional 5 participants were removed due to unknown dyspnoea severity/duration.
8. Symptoms were reported on a scale of 0-5, 0 for not present and 1-5 present with increasing severity. Fever was reported as present or not and was defined as temperatures above 38°C.
9. Respiratory symptoms exclude dyspnoea. Most participants reported more than one type of respiratory symptom during an infection episode, therefore we took the highest severity reported for any respiratory symptom. Accordingly, the duration was calculated from the day on which the first respiratory symptom was reported until the day the last respiratory symptom disappeared.
10. N=205: Includes only participants who experienced a SARS-CoV-2 infection and an additional 18 were removed due to unknown respiratory symptom severity.
11. N=200: Includes only participants who experienced a SARS-CoV-2 infection and an additional 23 were removed due to unknown or ongoing respiratory symptom duration.
12. N=217: Includes only participants who experienced a SARS-CoV-2 infection and an additional 6 were removed due to unknown presence of fever or unknown fever duration.
13. N=205: Includes only participants who experienced a SARS-CoV-2 infection and an additional 18 were removed due to unknown non-respiratory symptom severity.
14. Non-respiratory symptoms exclude fever. Most participants reported more than one type of non-respiratory symptom during an infection episode, and we therefore took the highest severity reported for any non-respiratory symptom. Accordingly, the duration was calculated from the day on which the first non-respiratory symptom appeared until the day the last non-respiratory symptom disappeared.

Figure S6: Sensitivity analyses: adding covariates to the final multivariable regression models

A. Multivariable model with M12 anti-S1 log_{10} concentrations as outcome[1]

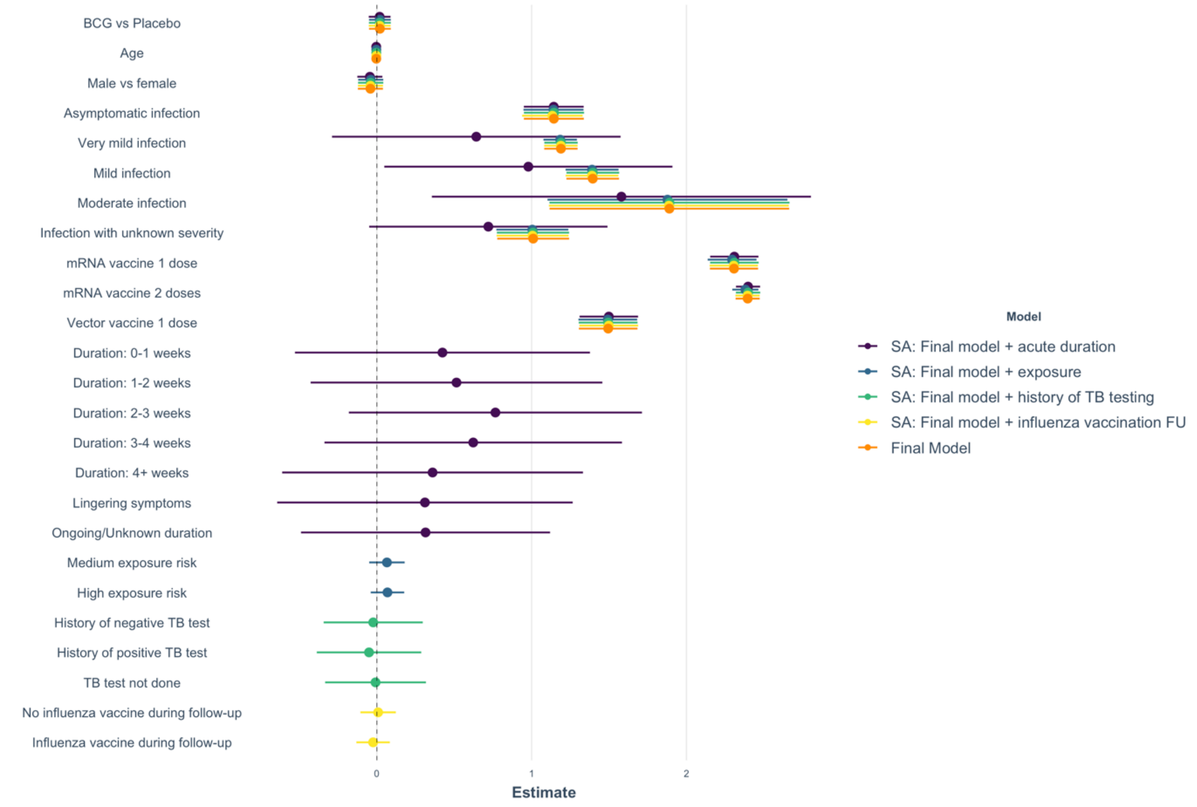

B. Multivariable model with M12 anti-N log_{10} concentrations as outcome[1]

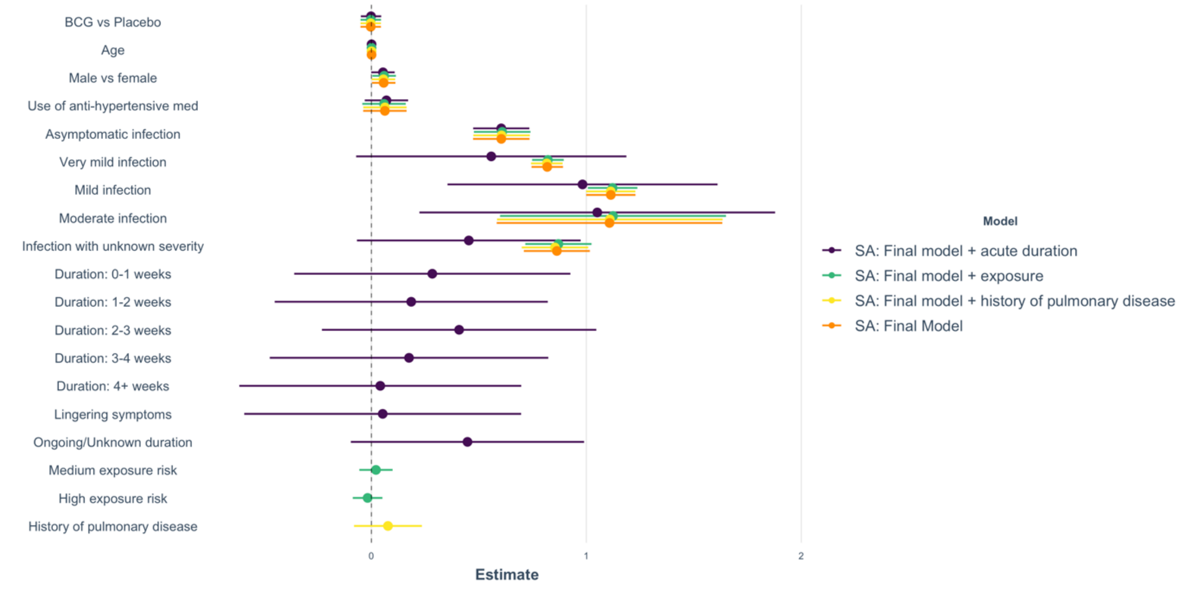

Abbreviations: BCG=Bacillus Calmette-Guérin; FU=follow up; med=medication; SA=sensitivity analysis; TB=tuberculosis.

1. The reference is an individual in the placebo group, female, who never experienced a SARS-CoV-2 infection and never received a single dose of a COVID-19 vaccine. Age is continuous (per year). The reference for workplace exposure risk is low risk. The reference for past TB test results is only having had negative TB test results.

Table S4: Sensitivity analyses: linear regression models after applying varying seroconversion windows

A: Multivariable model[1] with M12 anti-S1 log_{10} concentrations as outcome

Covariate	Seroconversion window = 14 days[3,4]		Seroconversion window = 7 days[3,5]		Seroconversion window = 0 days[3,6]	
	**Estimate (95% CI)**	**p-value**	**Estimate (95% CI)**	**p-value**	**Estimate (95% CI)**	**p-value**
Model intercept	0.50 (0.36, 0.64)	**<0.001**	0.47 (0.33, 0.61)	**<0.001**	0.44 (0.28, 0.60)	**<0.001**
BCG vs placebo[2]	0.02 (-0.05, 0.09)	0.585	0.06 (-0.01, 0.13)	0.118	0.02 (-0.06, 0.10)	0.627
Age (per year)	-0.004 (-0.01, -0.007)	**0.008**	-0.004 (-0.01, 0.001)	**0.009**	-0.004 (-0.01, -0.001)	**0.034**
Male sex	-0.04 (-0.12, 0.04)	0.311	-0.0 (-0.11, 0.06)	0.598	0.01 (-0.08, 0.11)	0.773
**All the below compared to participants who never had a SARS-CoV-2 infection or COVID-19 vaccination during follow-up:**						
Asymptomatic	1.14 (0.95, 1.34)	**<0.001**	1.07 (0.87, 1.27)	**<0.001**	1.11 (0.88, 1.33)	**<0.001**
Very mild	1.19 (1.08, 1.30)	**<0.001**	1.25 (1.14, 1.37)	**<0.001**	1.25 (1.13, 1.37)	**<0.001**
Mild	1.39 (1.22, 1.56)	**<0.001**	1.48 (1.31, 1.66)	**<0.001**	1.66 (1.47, 1.85)	**<0.001**
Moderate	1.89 (1.12, 2.66)	**<0.001**	2.16 (1.47, 2.84)	**<0.001**	2.71 (1.94, 3.48)	**<0.001**
Unknown severity	1.01 (0.78, 1.24)	**<0.001**	1.13 (0.88, 1.37)	**<0.001**	1.12 (0.85, 1.40)	**<0.001**
mRNA 1 dose	2.31 (2.15, 2.46)	**<0.001**	1.86 (1.71, 2.00)	**<0.001**	1.04 (0.91, 1.18)	**<0.001**
mRNA 2 doses	2.40 (2.32, 2.47)	**<0.001**	2.40 (2.31, 2.48)	**<0.001**	2.41 (2.32, 2.50)	**<0.001**
Vector 1 dose	1.49 (1.31, 1.68)	**<0.001**	1.28 (1.11, 1.45)	**<0.001**	0.87 (0.71, 1.03)	**<0.001**
**Model fit**	**R^2=0.84**		**R^2=0.81**		**R^2=0.76**	

B: Multivariable model[1] with M12 anti-N log_{10} concentrations as outcome

Covariate	Seroconversion window = 14 days[3,4]		Seroconversion window = 7 days[3,5]		Seroconversion window = 0 days[3,6]	
	**Estimate (95% CI)**	**p-value**	**Estimate (95% CI)**	**p-value**	**Estimate (95% CI)**	**p-value**
Model intercept	0.48 (0.39, 0.57)	**<0.001**	0.47 (0.38, 0.56)	**<0.001**	0.47 (0.38, 0.55)	**<0.001**
BCG vs placebo[2]	-0.003 (-0.05, 0.04)	0.901	-0.004 (-0.05, 0.05)	0.860	-0.004 (-0.05, 0.04)	0.876
Age (per year)	0.001 (-0.007, 0.003)	0.312	0.001 (-0.001, 0.003)	0.224	0.001 (-0.001, 0.003)	0.267
Male sex	0.06 (0.005, 0.11)	**0.040**	0.06 (0.01, 0.12)	**0.018**	0.06 (0.01, 0.12)	**0.019**
Hypertension med	0.06 (-0.04, 0.16)	0.222	0.05 (-0.04, 0.15)	0.284	0.05 (-0.04, 0.15)	0.287
**All the below compared to participants who never had a SARS-CoV-2 infection or COVID-19 vaccination during follow-up:**						
Asymptomatic	0.60 (0.47, 0.74)	**<0.001**	0.63 (0.50, 0.76)	**<0.001**	0.62 (0.49, 0.74)	**<0.001**
Very mild	0.82 (0.74, 0.89)	**<0.001**	0.82 (0.75, 0.89)	**<0.001**	0.81 (0.74, 0.87)	**<0.001**
Mild	1.11 (1.00, 1.23)	**<0.001**	1.07 (0.96, 1.18)	**<0.001**	1.07 (0.96, 1.18)	**<0.001**
Moderate	1.11 (0.58, 1.63)	**<0.001**	1.10 (0.67, 1.52)	**<0.001**	1.11 (0.68, 1.54)	**<0.001**
Unknown severity	0.86 (0.71, 1.02)	**<0.001**	0.88 (0.73, 1.03)	**<0.001**	0.88 (0.73, 1.03)	**<0.001**
**Model fit**	**R^2=0.49**		**R^2=0.49**		**R^2=0.48**	

Abbreviations: CI=Confidence interval; M=month; med=medication.

1. The reference is an individual in the placebo group, female, who never experienced a SARS-CoV-2 infection and never received a single dose of a COVID-19 vaccine.
2. BCG vs placebo vaccination at baseline was forced into the model to take the randomisation into account.
3. The VIF values for this model are all less than 5, which means that there is no evidence of multicollinearity.
4. Window period is 14 days. The model includes 970 participants and 223 infections. Four participants were in the seroconversion window for an infection and 125 for the first dose of a vaccine.
5. Window period is 7 days. The model includes 1,024 participants and 236 infections. Four participants were in the seroconversion window for an infection and 71 for the first dose of a vaccine.
6. Window period is 0 days. The model includes 1,099 participants and 249 infections.
